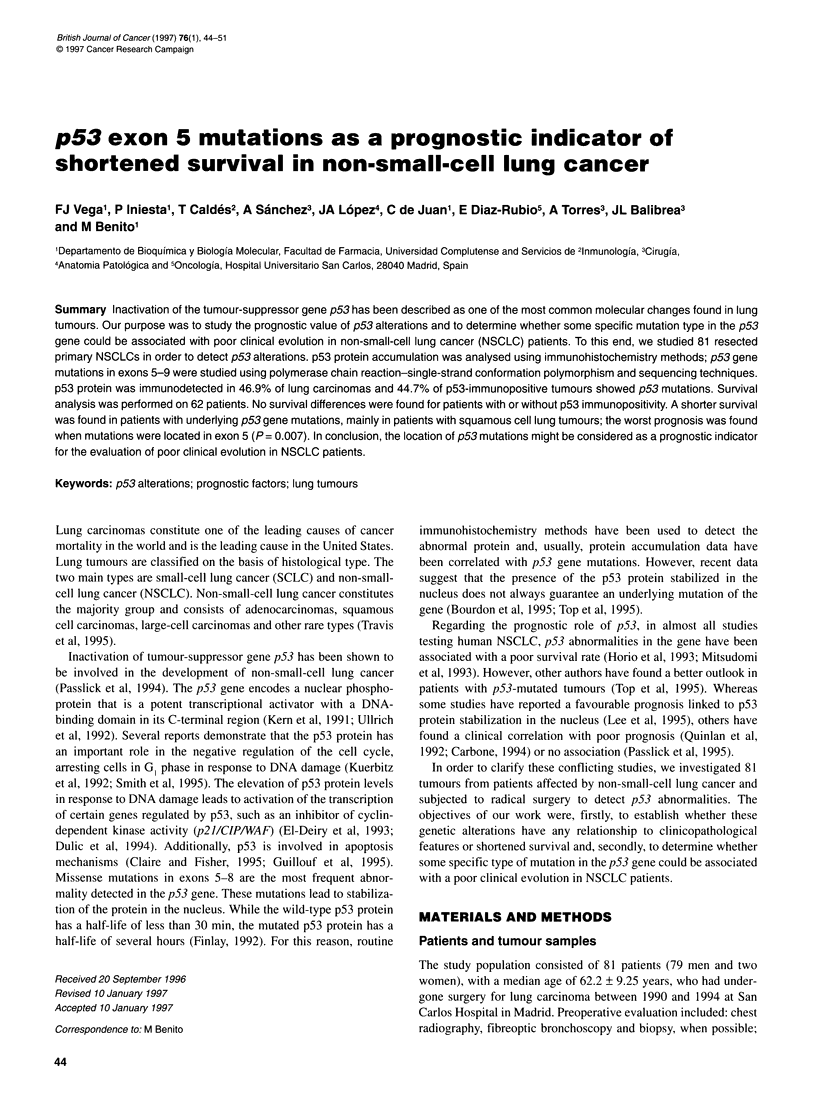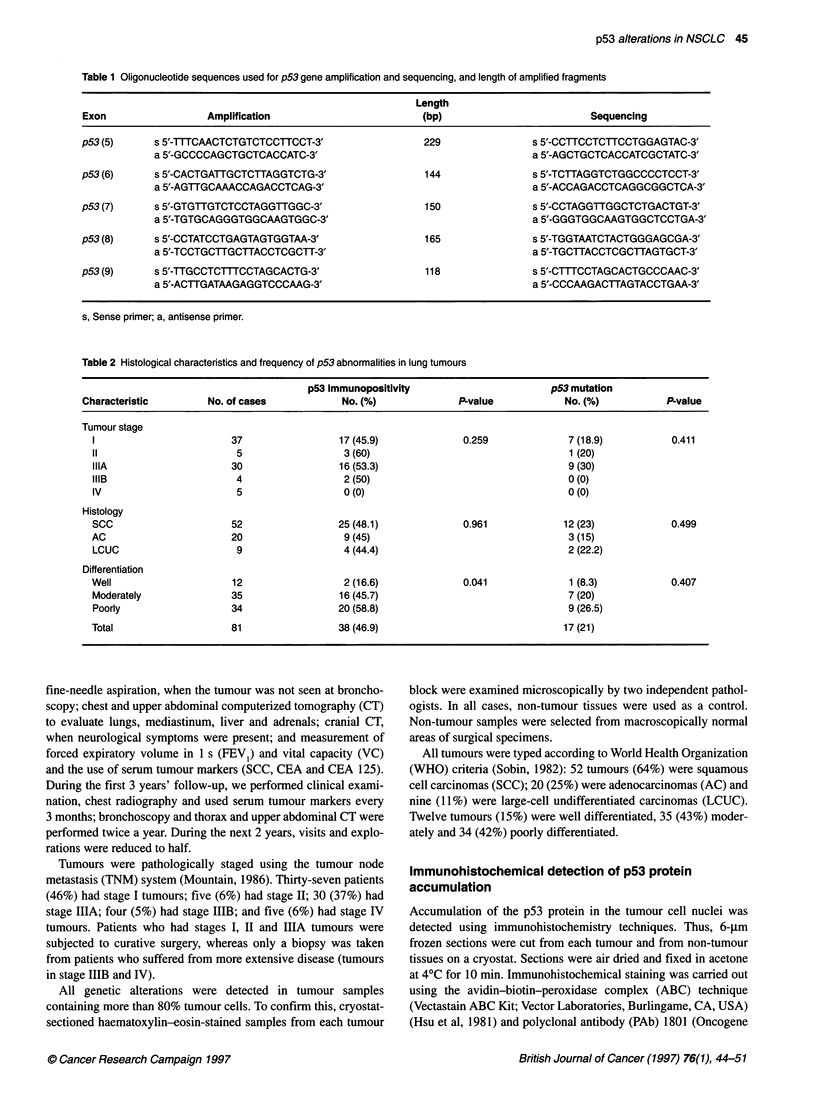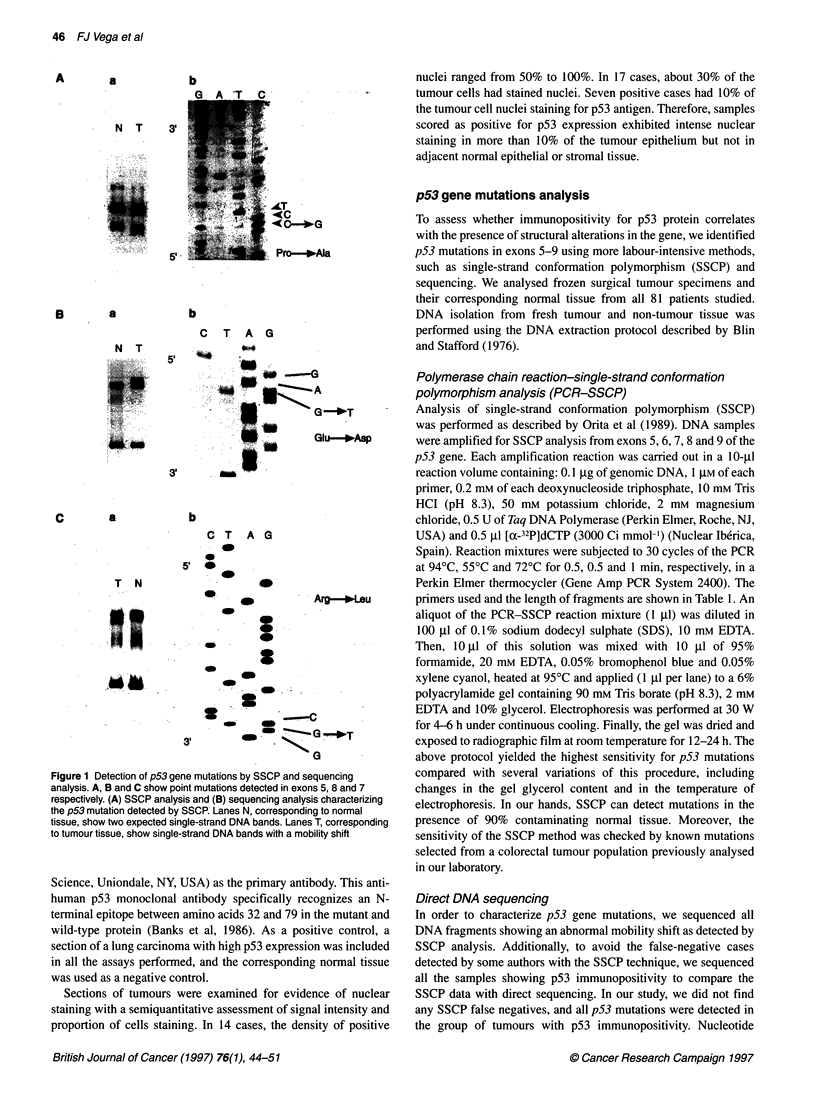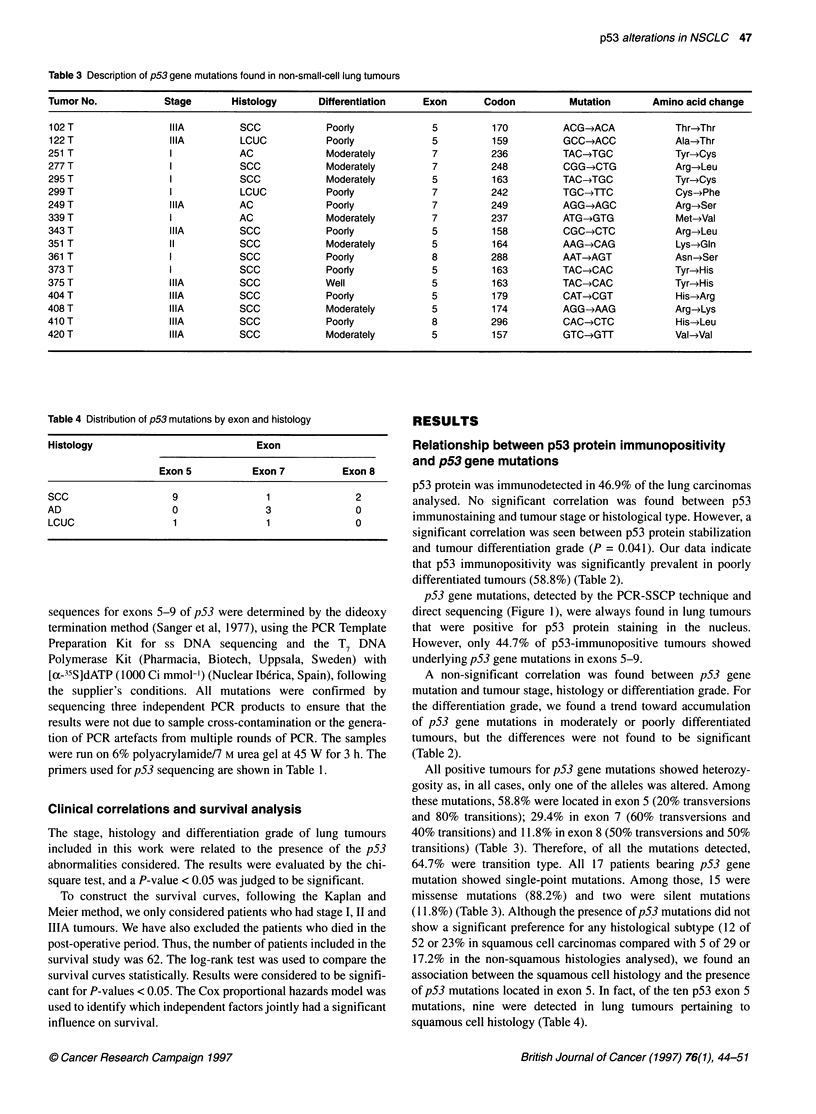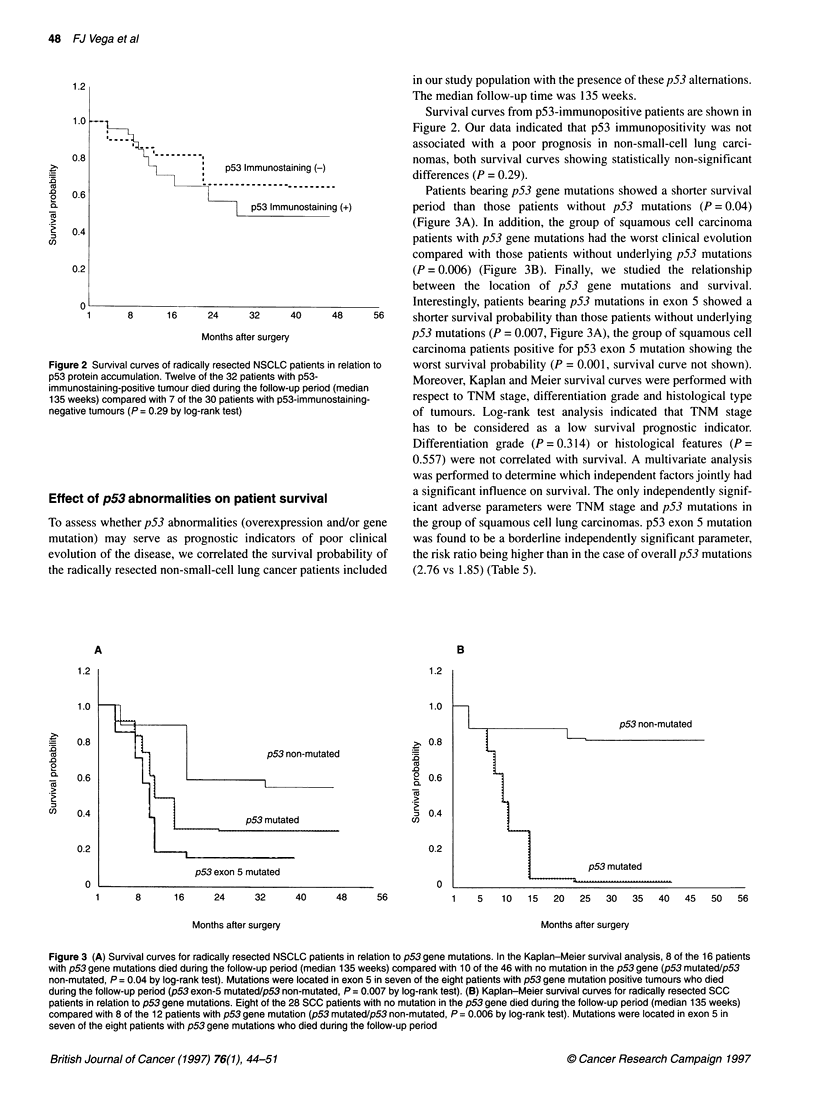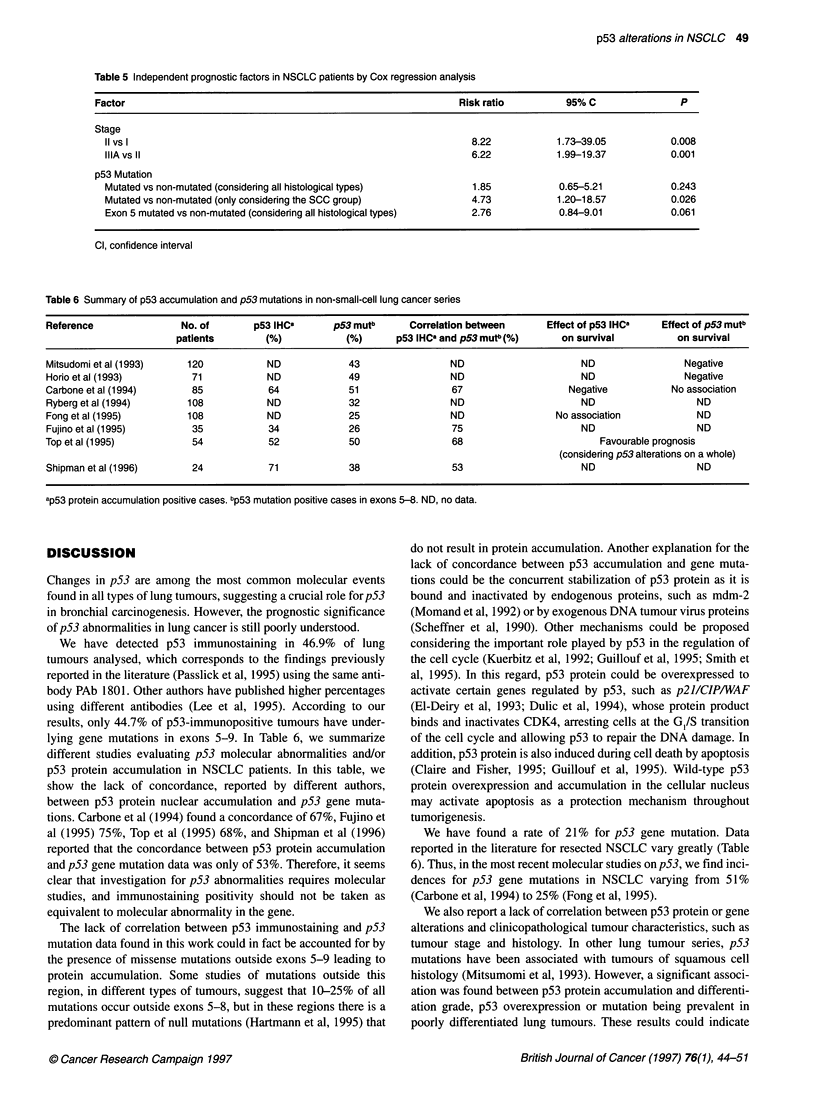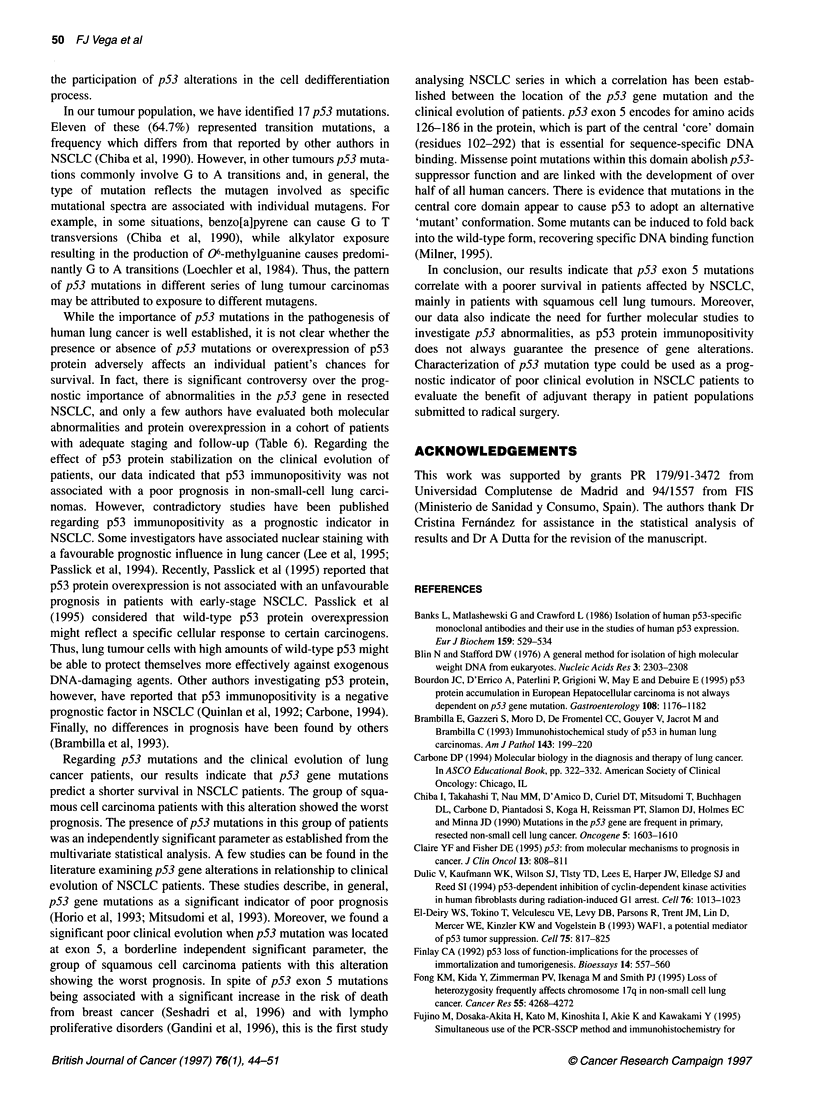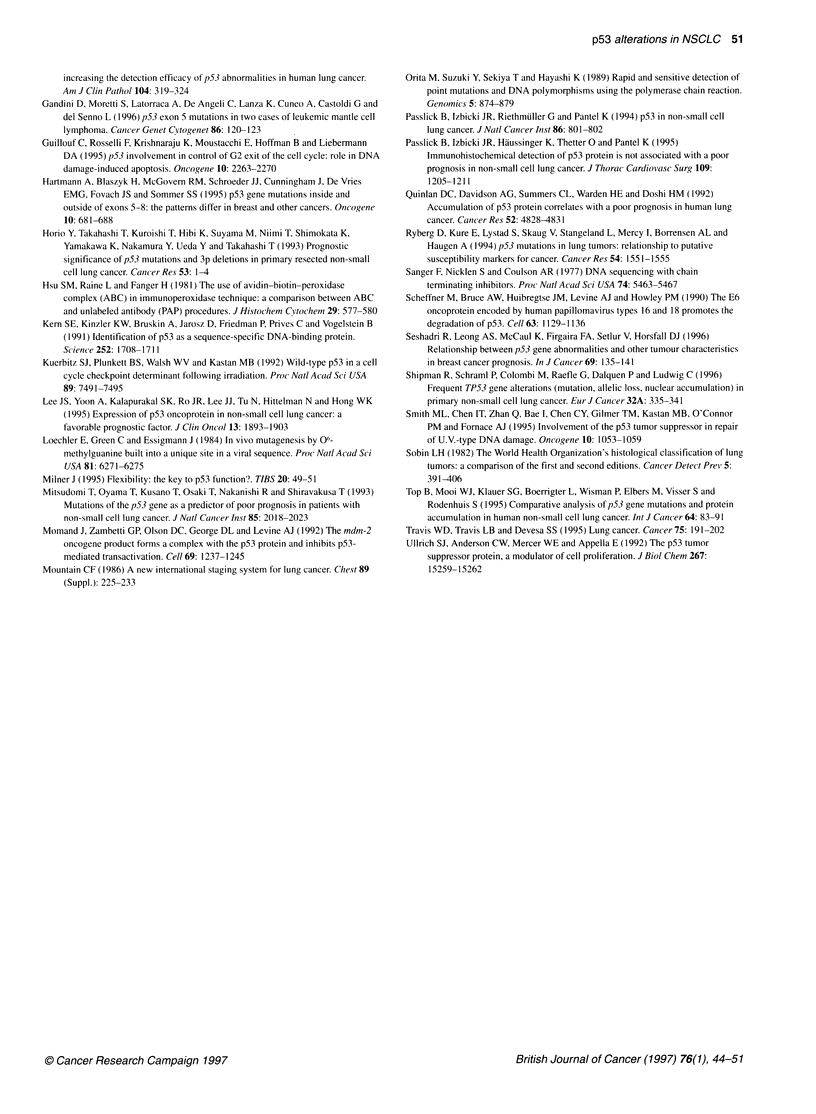# p53 exon 5 mutations as a prognostic indicator of shortened survival in non-small-cell lung cancer.

**DOI:** 10.1038/bjc.1997.334

**Published:** 1997

**Authors:** F. J. Vega, P. Iniesta, T. CaldÃ©s, A. Sanchez, J. A. LÃ³pez, C. de Juan, E. Diaz-Rubio, A. Torres, J. L. Balibrea, M. Benito

**Affiliations:** Departamento de Bioquimica y Biologia Molecular, Facultad de Farmacia, Universidad Complutense, Hospital Universitario San Carlos, Madrid, Spain.

## Abstract

**Images:**


British Journal of Cancer (1997) 76(1), 44–51
© 1997 Cancer Research Campaign

### *p53* exon 5 mutations as a prognostic indicator of shortened survival in non-small-cell lung cancer

**FJ Vega[1], P Iniesta[1], T Caldés[2], A Sánchez[3], JA López[4], C de Juan[1], E Diaz-Rubio[5], A Torres[3], JL Balibrea[3] and M Benito[1]**

[1]Departamento de Bioquímica y Biología Molecular, Facultad de Farmacia, Universidad Complutense and Servicios de [2]Inmunología, [3]Cirugía, [4]Anatomia Patológica and [5]Oncología, Hospital Universitario San Carlos, 28040 Madrid, Spain

**Summary** Inactivation of the tumour-suppressor gene *p53* has been described as one of the most common molecular changes found in lung tumours. Our purpose was to study the prognostic value of *p53* alterations and to determine whether some specific mutation type in the *p53* gene could be associated with poor clinical evolution in non-small-cell lung cancer (NSCLC) patients. To this end, we studied 81 resected primary NSCLCs in order to detect *p53* alterations. p53 protein accumulation was analysed using immunohistochemistry methods; *p53* gene mutations in exons 5–9 were studied using polymerase chain reaction–single-strand conformation polymorphism and sequencing techniques. p53 protein was immunodetected in 46.9% of lung carcinomas and 44.7% of p53-immunopositive tumours showed *p53* mutations. Survival analysis was performed on 62 patients. No survival differences were found for patients with or without p53 immunopositivity. A shorter survival was found in patients with underlying *p53* gene mutations, mainly in patients with squamous cell lung tumours; the worst prognosis was found when mutations were located in exon 5 ($P = 0.007$). In conclusion, the location of *p53* mutations might be considered as a prognostic indicator for the evaluation of poor clinical evolution in NSCLC patients.

**Keywords:** *p53* alterations; prognostic factors; lung tumours

Lung carcinomas constitute one of the leading causes of cancer mortality in the world and is the leading cause in the United States. Lung tumours are classified on the basis of histological type. The two main types are small-cell lung cancer (SCLC) and non-small-cell lung cancer (NSCLC). Non-small-cell lung cancer constitutes the majority group and consists of adenocarcinomas, squamous cell carcinomas, large-cell carcinomas and other rare types (Travis et al, 1995).

Inactivation of tumour-suppressor gene *p53* has been shown to be involved in the development of non-small-cell lung cancer (Passlick et al, 1994). The *p53* gene encodes a nuclear phosphoprotein that is a potent transcriptional activator with a DNA-binding domain in its C-terminal region (Kern et al, 1991; Ullrich et al, 1992). Several reports demonstrate that the p53 protein has an important role in the negative regulation of the cell cycle, arresting cells in $G_1$ phase in response to DNA damage (Kuerbitz et al, 1992; Smith et al, 1995). The elevation of p53 protein levels in response to DNA damage leads to activation of the transcription of certain genes regulated by p53, such as an inhibitor of cyclin-dependent kinase activity (*p21/CIP/WAF*) (El-Deiry et al, 1993; Dulic et al, 1994). Additionally, p53 is involved in apoptosis mechanisms (Claire and Fisher, 1995; Guillouf et al, 1995). Missense mutations in exons 5–8 are the most frequent abnormality detected in the *p53* gene. These mutations lead to stabilization of the protein in the nucleus. While the wild-type p53 protein has a half-life of less than 30 min, the mutated p53 protein has a half-life of several hours (Finlay, 1992). For this reason, routine immunohistochemistry methods have been used to detect the abnormal protein and, usually, protein accumulation data have been correlated with *p53* gene mutations. However, recent data suggest that the presence of the p53 protein stabilized in the nucleus does not always guarantee an underlying mutation of the gene (Bourdon et al, 1995; Top et al, 1995).

Regarding the prognostic role of *p53*, in almost all studies testing human NSCLC, *p53* abnormalities in the gene have been associated with a poor survival rate (Horio et al, 1993; Mitsudomi et al, 1993). However, other authors have found a better outlook in patients with *p53*-mutated tumours (Top et al, 1995). Whereas some studies have reported a favourable prognosis linked to p53 protein stabilization in the nucleus (Lee et al, 1995), others have found a clinical correlation with poor prognosis (Quinlan et al, 1992; Carbone, 1994) or no association (Passlick et al, 1995).

In order to clarify these conflicting studies, we investigated 81 tumours from patients affected by non-small-cell lung cancer and subjected to radical surgery to detect *p53* abnormalities. The objectives of our work were, firstly, to establish whether these genetic alterations have any relationship to clinicopathological features or shortened survival and, secondly, to determine whether some specific type of mutation in the *p53* gene could be associated with a poor clinical evolution in NSCLC patients.

### MATERIALS AND METHODS

### Patients and tumour samples

The study population consisted of 81 patients (79 men and two women), with a median age of 62.2 ± 9.25 years, who had undergone surgery for lung carcinoma between 1990 and 1994 at San Carlos Hospital in Madrid. Preoperative evaluation included: chest radiography, fibreoptic bronchoscopy and biopsy, when possible;

*Received 20 September 1996*
*Revised 10 January 1997*
*Accepted 10 January 1997*

*Correspondence to:* M Benito

**Table 1** Oligonucleotide sequences used for *p53* gene amplification and sequencing, and length of amplified fragments

| Exon | Amplification | Length (bp) | Sequencing |
|---|---|---|---|
| *p53* (5) | s 5′-TTTCAACTCTGTCTCCTTCCT-3′<br>a 5′-GCCCCAGCTGCTCACCATC-3′ | 229 | s 5′-CCTTCCTCTTCCTGGAGTAC-3′<br>a 5′-AGCTGCTCACCATCGCTATC-3′ |
| *p53* (6) | s 5′-CACTGATTGCTCTTAGGTCTG-3′<br>a 5′-AGTTGCAAACCAGACCTCAG-3′ | 144 | s 5′-TCTTAGGTCTGGCCCCTCCT-3′<br>a 5′-ACCAGACCTCAGGCGGCTCA-3′ |
| *p53* (7) | s 5′-GTGTTGTCTCCTAGGTTGGC-3′<br>a 5′-TGTGCAGGGTGGCAAGTGGC-3′ | 150 | s 5′-CCTAGGTTGGCTCTGACTGT-3′<br>a 5′-GGGTGGCAAGTGGCTCCTGA-3′ |
| *p53* (8) | s 5′-CCTATCCTGAGTAGTGGTAA-3′<br>a 5′-TCCTGCTTGCTTACCTCGCTT-3′ | 165 | s 5′-TGGTAATCTACTGGGAGCGA-3′<br>a 5′-TGCTTACCTCGCTTAGTGCT-3′ |
| *p53* (9) | s 5′-TTGCCTCTTTCCTAGCACTG-3′<br>a 5′-ACTTGATAAGAGGTCCCAAG-3′ | 118 | s 5′-CTTTCCTAGCACTGCCCAAC-3′<br>a 5′-CCCAAGACTTAGTACCTGAA-3′ |

s, Sense primer; a, antisense primer.

**Table 2** Histological characteristics and frequency of *p53* abnormalities in lung tumours

| Characteristic | No. of cases | p53 Immunopositivity No. (%) | *P*-value | *p53* mutation No. (%) | *P*-value |
|---|---|---|---|---|---|
| Tumour stage | | | | | |
| I | 37 | 17 (45.9) | 0.259 | 7 (18.9) | 0.411 |
| II | 5 | 3 (60) | | 1 (20) | |
| IIIA | 30 | 16 (53.3) | | 9 (30) | |
| IIIB | 4 | 2 (50) | | 0 (0) | |
| IV | 5 | 0 (0) | | 0 (0) | |
| Histology | | | | | |
| SCC | 52 | 25 (48.1) | 0.961 | 12 (23) | 0.499 |
| AC | 20 | 9 (45) | | 3 (15) | |
| LCUC | 9 | 4 (44.4) | | 2 (22.2) | |
| Differentiation | | | | | |
| Well | 12 | 2 (16.6) | 0.041 | 1 (8.3) | 0.407 |
| Moderately | 35 | 16 (45.7) | | 7 (20) | |
| Poorly | 34 | 20 (58.8) | | 9 (26.5) | |
| Total | 81 | 38 (46.9) | | 17 (21) | |

fine-needle aspiration, when the tumour was not seen at bronchoscopy; chest and upper abdominal computerized tomography (CT) to evaluate lungs, mediastinum, liver and adrenals; cranial CT, when neurological symptoms were present; and measurement of forced expiratory volume in 1 s ($FEV_1$) and vital capacity (VC) and the use of serum tumour markers (SCC, CEA and CEA 125). During the first 3 years' follow-up, we performed clinical examination, chest radiography and used serum tumour markers every 3 months; bronchoscopy and thorax and upper abdominal CT were performed twice a year. During the next 2 years, visits and explorations were reduced to half.

Tumours were pathologically staged using the tumour node metastasis (TNM) system (Mountain, 1986). Thirty-seven patients (46%) had stage I tumours; five (6%) had stage II; 30 (37%) had stage IIIA; four (5%) had stage IIIB; and five (6%) had stage IV tumours. Patients who had stages I, II and IIIA tumours were subjected to curative surgery, whereas only a biopsy was taken from patients who suffered from more extensive disease (tumours in stage IIIB and IV).

All genetic alterations were detected in tumour samples containing more than 80% tumour cells. To confirm this, cryostat-sectioned haematoxylin–eosin-stained samples from each tumour block were examined microscopically by two independent pathologists. In all cases, non-tumour tissues were used as a control. Non-tumour samples were selected from macroscopically normal areas of surgical specimens.

All tumours were typed according to World Health Organization (WHO) criteria (Sobin, 1982): 52 tumours (64%) were squamous cell carcinomas (SCC); 20 (25%) were adenocarcinomas (AC) and nine (11%) were large-cell undifferentiated carcinomas (LCUC). Twelve tumours (15%) were well differentiated, 35 (43%) moderately and 34 (42%) poorly differentiated.

### Immunohistochemical detection of p53 protein accumulation

Accumulation of the p53 protein in the tumour cell nuclei was detected using immunohistochemistry techniques. Thus, 6-μm frozen sections were cut from each tumour and from non-tumour tissues on a cryostat. Sections were air dried and fixed in acetone at 4°C for 10 min. Immunohistochemical staining was carried out using the avidin–biotin–peroxidase complex (ABC) technique (Vectastain ABC Kit; Vector Laboratories, Burlingame, CA, USA) (Hsu et al, 1981) and polyclonal antibody (PAb) 1801 (Oncogene

© Cancer Research Campaign 1997

**Figure 1** Detection of *p53* gene mutations by SSCP and sequencing analysis. **A**, **B** and **C** show point mutations detected in exons 5, 8 and 7 respectively. (**A**) SSCP analysis and (**B**) sequencing analysis characterizing the *p53* mutation detected by SSCP. Lanes N, corresponding to normal tissue, show two expected single-strand DNA bands. Lanes T, corresponding to tumour tissue, show single-strand DNA bands with a mobility shift

Science, Uniondale, NY, USA) as the primary antibody. This anti-human p53 monoclonal antibody specifically recognizes an N-terminal epitope between amino acids 32 and 79 in the mutant and wild-type protein (Banks et al, 1986). As a positive control, a section of a lung carcinoma with high p53 expression was included in all the assays performed, and the corresponding normal tissue was used as a negative control.

Sections of tumours were examined for evidence of nuclear staining with a semiquantitative assessment of signal intensity and proportion of cells staining. In 14 cases, the density of positive nuclei ranged from 50% to 100%. In 17 cases, about 30% of the tumour cells had stained nuclei. Seven positive cases had 10% of the tumour cell nuclei staining for p53 antigen. Therefore, samples scored as positive for p53 expression exhibited intense nuclear staining in more than 10% of the tumour epithelium but not in adjacent normal epithelial or stromal tissue.

### *p53* gene mutations analysis

To assess whether immunopositivity for p53 protein correlates with the presence of structural alterations in the gene, we identified *p53* mutations in exons 5–9 using more labour-intensive methods, such as single-strand conformation polymorphism (SSCP) and sequencing. We analysed frozen surgical tumour specimens and their corresponding normal tissue from all 81 patients studied. DNA isolation from fresh tumour and non-tumour tissue was performed using the DNA extraction protocol described by Blin and Stafford (1976).

### *Polymerase chain reaction–single-strand conformation polymorphism analysis (PCR–SSCP)*

Analysis of single-strand conformation polymorphism (SSCP) was performed as described by Orita et al (1989). DNA samples were amplified for SSCP analysis from exons 5, 6, 7, 8 and 9 of the *p53* gene. Each amplification reaction was carried out in a 10-µl reaction volume containing: 0.1 µg of genomic DNA, 1 µM of each primer, 0.2 mM of each deoxynucleoside triphosphate, 10 mM Tris HCI (pH 8.3), 50 mM potassium chloride, 2 mM magnesium chloride, 0.5 U of *Taq* DNA Polymerase (Perkin Elmer, Roche, NJ, USA) and 0.5 µl [α-$^{32}$P]dCTP (3000 Ci mmol$^{-1}$) (Nuclear Ibérica, Spain). Reaction mixtures were subjected to 30 cycles of the PCR at 94°C, 55°C and 72°C for 0.5, 0.5 and 1 min, respectively, in a Perkin Elmer thermocycler (Gene Amp PCR System 2400). The primers used and the length of fragments are shown in Table 1. An aliquot of the PCR–SSCP reaction mixture (1 µl) was diluted in 100 µl of 0.1% sodium dodecyl sulphate (SDS), 10 mM EDTA. Then, 10 µl of this solution was mixed with 10 µl of 95% formamide, 20 mM EDTA, 0.05% bromophenol blue and 0.05% xylene cyanol, heated at 95°C and applied (1 µl per lane) to a 6% polyacrylamide gel containing 90 mM Tris borate (pH 8.3), 2 mM EDTA and 10% glycerol. Electrophoresis was performed at 30 W for 4–6 h under continuous cooling. Finally, the gel was dried and exposed to radiographic film at room temperature for 12–24 h. The above protocol yielded the highest sensitivity for *p53* mutations compared with several variations of this procedure, including changes in the gel glycerol content and in the temperature of electrophoresis. In our hands, SSCP can detect mutations in the presence of 90% contaminating normal tissue. Moreover, the sensitivity of the SSCP method was checked by known mutations selected from a colorectal tumour population previously analysed in our laboratory.

### *Direct DNA sequencing*

In order to characterize *p53* gene mutations, we sequenced all DNA fragments showing an abnormal mobility shift as detected by SSCP analysis. Additionally, to avoid the false-negative cases detected by some authors with the SSCP technique, we sequenced all the samples showing p53 immunopositivity to compare the SSCP data with direct sequencing. In our study, we did not find any SSCP false negatives, and all *p53* mutations were detected in the group of tumours with p53 immunopositivity. Nucleotide

© Cancer Research Campaign 1997

**Table 3** Description of *p53* gene mutations found in non-small-cell lung tumours

| Tumor No. | Stage | Histology | Differentiation | Exon | Codon | Mutation | Amino acid change |
|---|---|---|---|---|---|---|---|
| 102 T | IIIA | SCC | Poorly | 5 | 170 | ACG→ACA | Thr→Thr |
| 122 T | IIIA | LCUC | Poorly | 5 | 159 | GCC→ACC | Ala→Thr |
| 251 T | I | AC | Moderately | 7 | 236 | TAC→TGC | Tyr→Cys |
| 277 T | I | SCC | Moderately | 7 | 248 | CGG→CTG | Arg→Leu |
| 295 T | I | SCC | Moderately | 5 | 163 | TAC→TGC | Tyr→Cys |
| 299 T | I | LCUC | Poorly | 7 | 242 | TGC→TTC | Cys→Phe |
| 249 T | IIIA | AC | Poorly | 7 | 249 | AGG→AGC | Arg→Ser |
| 339 T | I | AC | Moderately | 7 | 237 | ATG→GTG | Met→Val |
| 343 T | IIIA | SCC | Poorly | 5 | 158 | CGC→CTC | Arg→Leu |
| 351 T | II | SCC | Moderately | 5 | 164 | AAG→CAG | Lys→Gln |
| 361 T | I | SCC | Poorly | 8 | 288 | AAT→AGT | Asn→Ser |
| 373 T | I | SCC | Poorly | 5 | 163 | TAC→CAC | Tyr→His |
| 375 T | IIIA | SCC | Well | 5 | 163 | TAC→CAC | Tyr→His |
| 404 T | IIIA | SCC | Poorly | 5 | 179 | CAT→CGT | His→Arg |
| 408 T | IIIA | SCC | Moderately | 5 | 174 | AGG→AAG | Arg→Lys |
| 410 T | IIIA | SCC | Poorly | 8 | 296 | CAC→CTC | His→Leu |
| 420 T | IIIA | SCC | Moderately | 5 | 157 | GTC→GTT | Val→Val |

**Table 4** Distribution of *p53* mutations by exon and histology

| Histology | Exon | | |
|---|---|---|---|
| | Exon 5 | Exon 7 | Exon 8 |
| SCC | 9 | 1 | 2 |
| AD | 0 | 3 | 0 |
| LCUC | 1 | 1 | 0 |

sequences for exons 5–9 of *p53* were determined by the dideoxy termination method (Sanger et al, 1977), using the PCR Template Preparation Kit for ss DNA sequencing and the $T_7$ DNA Polymerase Kit (Pharmacia, Biotech, Uppsala, Sweden) with [$\alpha$-$^{35}$S]dATP (1000 Ci mmol$^{-1}$) (Nuclear Ibérica, Spain), following the supplier's conditions. All mutations were confirmed by sequencing three independent PCR products to ensure that the results were not due to sample cross-contamination or the generation of PCR artefacts from multiple rounds of PCR. The samples were run on 6% polyacrylamide/7 M urea gel at 45 W for 3 h. The primers used for *p53* sequencing are shown in Table 1.

### Clinical correlations and survival analysis

The stage, histology and differentiation grade of lung tumours included in this work were related to the presence of the *p53* abnormalities considered. The results were evaluated by the chi-square test, and a *P*-value < 0.05 was judged to be significant.

To construct the survival curves, following the Kaplan and Meier method, we only considered patients who had stage I, II and IIIA tumours. We have also excluded the patients who died in the post-operative period. Thus, the number of patients included in the survival study was 62. The log-rank test was used to compare the survival curves statistically. Results were considered to be significant for *P*-values < 0.05. The Cox proportional hazards model was used to identify which independent factors jointly had a significant influence on survival.

### RESULTS

### Relationship between p53 protein immunopositivity and *p53* gene mutations p53 protein was immunodetected in 46.9% of the lung carcinomas analysed. No significant correlation was found between p53 immunostaining and tumour stage or histological type. However, a significant correlation was seen between p53 protein stabilization and tumour differentiation grade ($P$ = 0.041). Our data indicate that p53 immunopositivity was significantly prevalent in poorly differentiated tumours (58.8%) (Table 2).

*p53* gene mutations, detected by the PCR-SSCP technique and direct sequencing (Figure 1), were always found in lung tumours that were positive for p53 protein staining in the nucleus. However, only 44.7% of p53-immunopositive tumours showed underlying *p53* gene mutations in exons 5–9.

A non-significant correlation was found between *p53* gene mutation and tumour stage, histology or differentiation grade. For the differentiation grade, we found a trend toward accumulation of *p53* gene mutations in moderately or poorly differentiated tumours, but the differences were not found to be significant (Table 2).

All positive tumours for *p53* gene mutations showed heterozygosity as, in all cases, only one of the alleles was altered. Among these mutations, 58.8% were located in exon 5 (20% transversions and 80% transitions); 29.4% in exon 7 (60% transversions and 40% transitions) and 11.8% in exon 8 (50% transversions and 50% transitions) (Table 3). Therefore, of all the mutations detected, 64.7% were transition type. All 17 patients bearing *p53* gene mutation showed single-point mutations. Among those, 15 were missense mutations (88.2%) and two were silent mutations (11.8%) (Table 3). Although the presence of *p53* mutations did not show a significant preference for any histological subtype (12 of 52 or 23% in squamous cell carcinomas compared with 5 of 29 or 17.2% in the non-squamous histologies analysed), we found an association between the squamous cell histology and the presence of *p53* mutations located in exon 5. In fact, of the ten p53 exon 5 mutations, nine were detected in lung tumours pertaining to squamous cell histology (Table 4).

© Cancer Research Campaign 1997

**Figure 2** Survival curves of radically resected NSCLC patients in relation to p53 protein accumulation. Twelve of the 32 patients with p53-immunostaining-positive tumour died during the follow-up period (median 135 weeks) compared with 7 of the 30 patients with p53-immunostaining-negative tumours ($P = 0.29$ by log-rank test)

### Effect of *p53* abnormalities on patient survival

To assess whether *p53* abnormalities (overexpression and/or gene mutation) may serve as prognostic indicators of poor clinical evolution of the disease, we correlated the survival probability of the radically resected non-small-cell lung cancer patients included in our study population with the presence of these *p53* alternations. The median follow-up time was 135 weeks.

Survival curves from p53-immunopositive patients are shown in Figure 2. Our data indicated that p53 immunopositivity was not associated with a poor prognosis in non-small-cell lung carcinomas, both survival curves showing statistically non-significant differences ($P = 0.29$).

Patients bearing *p53* gene mutations showed a shorter survival period than those patients without *p53* mutations ($P = 0.04$) (Figure 3A). In addition, the group of squamous cell carcinoma patients with *p53* gene mutations had the worst clinical evolution compared with those patients without underlying *p53* mutations ($P = 0.006$) (Figure 3B). Finally, we studied the relationship between the location of *p53* gene mutations and survival. Interestingly, patients bearing *p53* mutations in exon 5 showed a shorter survival probability than those patients without underlying *p53* mutations ($P = 0.007$, Figure 3A), the group of squamous cell carcinoma patients positive for p53 exon 5 mutation showing the worst survival probability ($P = 0.001$, survival curve not shown). Moreover, Kaplan and Meier survival curves were performed with respect to TNM stage, differentiation grade and histological type of tumours. Log-rank test analysis indicated that TNM stage has to be considered as a low survival prognostic indicator. Differentiation grade ($P = 0.314$) or histological features ($P = 0.557$) were not correlated with survival. A multivariate analysis was performed to determine which independent factors jointly had a significant influence on survival. The only independently significant adverse parameters were TNM stage and *p53* mutations in the group of squamous cell lung carcinomas. p53 exon 5 mutation was found to be a borderline independently significant parameter, the risk ratio being higher than in the case of overall *p53* mutations (2.76 vs 1.85) (Table 5).

**Figure 3** (**A**) Survival curves for radically resected NSCLC patients in relation to *p53* gene mutations. In the Kaplan–Meier survival analysis, 8 of the 16 patients with *p53* gene mutations died during the follow-up period (median 135 weeks) compared with 10 of the 46 with no mutation in the *p53* gene (*p53* mutated/*p53* non-mutated, $P = 0.04$ by log-rank test). Mutations were located in exon 5 in seven of the eight patients with *p53* gene mutation positive tumours who died during the follow-up period (*p53* exon-5 mutated/*p53* non-mutated, $P = 0.007$ by log-rank test). (**B**) Kaplan–Meier survival curves for radically resected SCC patients in relation to *p53* gene mutations. Eight of the 28 SCC patients with no mutation in the *p53* gene died during the follow-up period (median 135 weeks) compared with 8 of the 12 patients with *p53* gene mutation (*p53* mutated/*p53* non-mutated, $P = 0.006$ by log-rank test). Mutations were located in exon 5 in seven of the eight patients with *p53* gene mutations who died during the follow-up period

© Cancer Research Campaign 1997

**Table 5** Independent prognostic factors in NSCLC patients by Cox regression analysis

| Factor | Risk ratio | 95% C | P |
|---|---|---|---|
| Stage | | | |
| II vs I | 8.22 | 1.73–39.05 | 0.008 |
| IIIA vs II | 6.22 | 1.99–19.37 | 0.001 |
| p53 Mutation | | | |
| Mutated vs non-mutated (considering all histological types) | 1.85 | 0.65–5.21 | 0.243 |
| Mutated vs non-mutated (only considering the SCC group) | 4.73 | 1.20–18.57 | 0.026 |
| Exon 5 mutated vs non-mutated (considering all histological types) | 2.76 | 0.84–9.01 | 0.061 |

CI, confidence interval

**Table 6** Summary of p53 accumulation and *p53* mutations in non-small-cell lung cancer series

| Reference | No. of patients | p53 IHC[a] (%) | *p53* mut[b] (%) | Correlation between p53 IHC[a] and *p53* mut[b] (%) | Effect of p53 IHC[a] on survival | Effect of *p53* mut[b] on survival |
|---|---|---|---|---|---|---|
| Mitsudomi et al (1993) | 120 | ND | 43 | ND | ND | Negative |
| Horio et al (1993) | 71 | ND | 49 | ND | ND | Negative |
| Carbone et al (1994) | 85 | 64 | 51 | 67 | Negative | No association |
| Ryberg et al (1994) | 108 | ND | 32 | ND | ND | ND |
| Fong et al (1995) | 108 | ND | 25 | ND | No association | ND |
| Fujino et al (1995) | 35 | 34 | 26 | 75 | ND | ND |
| Top et al (1995) | 54 | 52 | 50 | 68 | Favourable prognosis (considering *p53* alterations on a whole) | |
| Shipman et al (1996) | 24 | 71 | 38 | 53 | ND | ND |

[a]p53 protein accumulation positive cases. [b]p53 mutation positive cases in exons 5–8. ND, no data.

### DISCUSSION

Changes in *p53* are among the most common molecular events found in all types of lung tumours, suggesting a crucial role for *p53* in bronchial carcinogenesis. However, the prognostic significance of *p53* abnormalities in lung cancer is still poorly understood.

We have detected p53 immunostaining in 46.9% of lung tumours analysed, which corresponds to the findings previously reported in the literature (Passlick et al, 1995) using the same antibody PAb 1801. Other authors have published higher percentages using different antibodies (Lee et al, 1995). According to our results, only 44.7% of p53-immunopositive tumours have underlying gene mutations in exons 5–9. In Table 6, we summarize different studies evaluating *p53* molecular abnormalities and/or p53 protein accumulation in NSCLC patients. In this table, we show the lack of concordance, reported by different authors, between p53 protein nuclear accumulation and *p53* gene mutations. Carbone et al (1994) found a concordance of 67%, Fujino et al (1995) 75%, Top et al (1995) 68%, and Shipman et al (1996) reported that the concordance between p53 protein accumulation and *p53* gene mutation data was only of 53%. Therefore, it seems clear that investigation for *p53* abnormalities requires molecular studies, and immunostaining positivity should not be taken as equivalent to molecular abnormality in the gene.

The lack of correlation between p53 immunostaining and *p53* mutation data found in this work could in fact be accounted for by the presence of missense mutations outside exons 5–9 leading to protein accumulation. Some studies of mutations outside this region, in different types of tumours, suggest that 10–25% of all mutations occur outside exons 5–8, but in these regions there is a predominant pattern of null mutations (Hartmann et al, 1995) that do not result in protein accumulation. Another explanation for the lack of concordance between p53 accumulation and gene mutations could be the concurrent stabilization of p53 protein as it is bound and inactivated by endogenous proteins, such as mdm-2 (Momand et al, 1992) or by exogenous DNA tumour virus proteins (Scheffner et al, 1990). Other mechanisms could be proposed considering the important role played by p53 in the regulation of the cell cycle (Kuerbitz et al, 1992; Guillouf et al, 1995; Smith et al, 1995). In this regard, p53 protein could be overexpressed to activate certain genes regulated by p53, such as *p21/CIP/WAF* (El-Deiry et al, 1993; Dulic et al, 1994), whose protein product binds and inactivates CDK4, arresting cells at the $G_1$/S transition of the cell cycle and allowing p53 to repair the DNA damage. In addition, p53 protein is also induced during cell death by apoptosis (Claire and Fisher, 1995; Guillouf et al, 1995). Wild-type p53 protein overexpression and accumulation in the cellular nucleus may activate apoptosis as a protection mechanism throughout tumorigenesis.

We have found a rate of 21% for *p53* gene mutation. Data reported in the literature for resected NSCLC vary greatly (Table 6). Thus, in the most recent molecular studies on *p53*, we find incidences for *p53* gene mutations in NSCLC varying from 51% (Carbone et al, 1994) to 25% (Fong et al, 1995).

We also report a lack of correlation between p53 protein or gene alterations and clinicopathological tumour characteristics, such as tumour stage and histology. In other lung tumour series, *p53* mutations have been associated with tumours of squamous cell histology (Mitsumomi et al, 1993). However, a significant association was found between p53 protein accumulation and differentiation grade, p53 overexpression or mutation being prevalent in poorly differentiated lung tumours. These results could indicate

© *Cancer Research Campaign 1997* 

the participation of *p53* alterations in the cell dedifferentiation process.

In our tumour population, we have identified 17 *p53* mutations. Eleven of these (64.7%) represented transition mutations, a frequency which differs from that reported by other authors in NSCLC (Chiba et al, 1990). However, in other tumours *p53* mutations commonly involve G to A transitions and, in general, the type of mutation reflects the mutagen involved as specific mutational spectra are associated with individual mutagens. For example, in some situations, benzo[a]pyrene can cause G to T transversions (Chiba et al, 1990), while alkylator exposure resulting in the production of $O^6$-methylguanine causes predominantly G to A transitions (Loechler et al, 1984). Thus, the pattern of *p53* mutations in different series of lung tumour carcinomas may be attributed to exposure to different mutagens.

While the importance of *p53* mutations in the pathogenesis of human lung cancer is well established, it is not clear whether the presence or absence of *p53* mutations or overexpression of p53 protein adversely affects an individual patient's chances for survival. In fact, there is significant controversy over the prognostic importance of abnormalities in the *p53* gene in resected NSCLC, and only a few authors have evaluated both molecular abnormalities and protein overexpression in a cohort of patients with adequate staging and follow-up (Table 6). Regarding the effect of p53 protein stabilization on the clinical evolution of patients, our data indicated that p53 immunopositivity was not associated with a poor prognosis in non-small-cell lung carcinomas. However, contradictory studies have been published regarding p53 immunopositivity as a prognostic indicator in NSCLC. Some investigators have associated nuclear staining with a favourable prognostic influence in lung cancer (Lee et al, 1995; Passlick et al, 1994). Recently, Passlick et al (1995) reported that p53 protein overexpression is not associated with an unfavourable prognosis in patients with early-stage NSCLC. Passlick et al (1995) considered that wild-type p53 protein overexpression might reflect a specific cellular response to certain carcinogens. Thus, lung tumour cells with high amounts of wild-type p53 might be able to protect themselves more effectively against exogenous DNA-damaging agents. Other authors investigating p53 protein, however, have reported that p53 immunopositivity is a negative prognostic factor in NSCLC (Quinlan et al, 1992; Carbone, 1994). Finally, no differences in prognosis have been found by others (Brambilla et al, 1993).

Regarding *p53* mutations and the clinical evolution of lung cancer patients, our results indicate that *p53* gene mutations predict a shorter survival in NSCLC patients. The group of squamous cell carcinoma patients with this alteration showed the worst prognosis. The presence of *p53* mutations in this group of patients was an independently significant parameter as established from the multivariate statistical analysis. A few studies can be found in the literature examining *p53* gene alterations in relationship to clinical evolution of NSCLC patients. These studies describe, in general, *p53* gene mutations as a significant indicator of poor prognosis (Horio et al, 1993; Mitsudomi et al, 1993). Moreover, we found a significant poor clinical evolution when *p53* mutation was located at exon 5, a borderline independent significant parameter, the group of squamous cell carcinoma patients with this alteration showing the worst prognosis. In spite of *p53* exon 5 mutations being associated with a significant increase in the risk of death from breast cancer (Seshadri et al, 1996) and with lympho proliferative disorders (Gandini et al, 1996), this is the first study analysing NSCLC series in which a correlation has been established between the location of the *p53* gene mutation and the clinical evolution of patients. *p53* exon 5 encodes for amino acids 126–186 in the protein, which is part of the central 'core' domain (residues 102–292) that is essential for sequence-specific DNA binding. Missense point mutations within this domain abolish *p53*-suppressor function and are linked with the development of over half of all human cancers. There is evidence that mutations in the central core domain appear to cause p53 to adopt an alternative 'mutant' conformation. Some mutants can be induced to fold back into the wild-type form, recovering specific DNA binding function (Milner, 1995).

In conclusion, our results indicate that *p53* exon 5 mutations correlate with a poorer survival in patients affected by NSCLC, mainly in patients with squamous cell lung tumours. Moreover, our data also indicate the need for further molecular studies to investigate *p53* abnormalities, as p53 protein immunopositivity does not always guarantee the presence of gene alterations. Characterization of *p53* mutation type could be used as a prognostic indicator of poor clinical evolution in NSCLC patients to evaluate the benefit of adjuvant therapy in patient populations submitted to radical surgery.

### ACKNOWLEDGEMENTS

This work was supported by grants PR 179/91-3472 from Universidad Complutense de Madrid and 94/1557 from FIS (Ministerio de Sanidad y Consumo, Spain). The authors thank Dr Cristina Fernández for assistance in the statistical analysis of results and Dr A Dutta for the revision of the manuscript.

### REFERENCES

Banks L, Matlashewski G and Crawford L (1986) Isolation of human p53-specific monoclonal antibodies and their use in the studies of human p53 expression. *Eur J Biochem* **159**: 529–534

Blin N and Stafford DW (1976) A general method for isolation of high molecular weight DNA from eukaryotes. *Nucleic Acids Res* **3**: 2303–2308

Bourdon JC, D'Errico A, Paterlini P, Grigioni W, May E and Debuire E (1995) p53 protein accumulation in European Hepatocellular carcinoma is not always dependent on *p53* gene mutation. *Gastroenterology* **108**: 1176–1182

Brambilla E, Gazzeri S, Moro D, De Fromentel CC, Gouyer V, Jacrot M and Brambilla C (1993) Immunohistochemical study of p53 in human lung carcinomas. *Am J Pathol* **143**: 199–220

Carbone DP (1994) Molecular biology in the diagnosis and therapy of lung cancer. In *ASCO Educational Book*, pp. 322–332. American Society of Clinical Oncology: Chicago, IL

Chiba I, Takahashi T, Nau MM, D'Amico D, Curiel DT, Mitsudomi T, Buchhagen DL, Carbone D, Piantadosi S, Koga H, Reissman PT, Slamon DJ, Holmes EC and Minna JD (1990) Mutations in the *p53* gene are frequent in primary, resected non-small cell lung cancer. *Oncogene* **5**: 1603–1610

Claire YF and Fisher DE (1995) *p53*: from molecular mechanisms to prognosis in cancer. *J Clin Oncol* **13**: 808–811

Dulic V, Kaufmann WK, Wilson SJ, Tlsty TD, Lees E, Harper JW, Elledge SJ and Reed SI (1994) p53-dependent inhibition of cyclin-dependent kinase activities in human fibroblasts during radiation-induced G1 arrest. *Cell* **76**: 1013–1023

El-Deiry WS, Tokino T, Velculescu VE, Levy DB, Parsons R, Trent JM, Lin D, Mercer WE, Kinzler KW and Vogelstein B (1993) WAF1, a potential mediator of p53 tumor suppression. *Cell* **75**: 817–825

Finlay CA (1992) p53 loss of function-implications for the processes of immortalization and tumorigenesis. *Bioessays* **14**: 557–560

Fong KM, Kida Y, Zimmerman PV, Ikenaga M and Smith PJ (1995) Loss of heterozygosity frequently affects chromosome 17q in non-small cell lung cancer. *Cancer Res* **55**: 4268–4272

Fujino M, Dosaka-Akita H, Kato M, Kinoshita I, Akie K and Kawakami Y (1995) Simultaneous use of the PCR-SSCP method and immunohistochemistry for

© Cancer Research Campaign 1997

increasing the detection efficacy of *p53* abnormalities in human lung cancer. *Am J Clin Pathol* **104**: 319–324

Gandini D, Moretti S, Latorraca A, De Angeli C, Lanza K, Cuneo A, Castoldi G and del Senno L (1996) *p53* exon 5 mutations in two cases of leukemic mantle cell lymphoma. *Cancer Genet Cytogenet* **86**: 120–123

Guillouf C, Rosselli F, Krishnaraju K, Moustacchi E, Hoffman B and Liebermann DA (1995) *p53* involvement in control of G2 exit of the cell cycle: role in DNA damage-induced apoptosis. *Oncogene* **10**: 2263–2270

Hartmann A, Blaszyk H, McGovern RM, Schroeder JJ, Cunningham J, De Vries EMG, Fovach JS and Sommer SS (1995) p53 gene mutations inside and outside of exons 5–8: the patterns differ in breast and other cancers. *Oncogene* **10**: 681–688

Horio Y, Takahashi T, Kuroishi T, Hibi K, Suyama M, Niimi T, Shimokata K, Yamakawa K, Nakamura Y, Ueda Y and Takahashi T (1993) Prognostic significance of *p53* mutations and 3p deletions in primary resected non-small cell lung cancer. *Cancer Res* **53**: 1–4

Hsu SM, Raine L and Fanger H (1981) The use of avidin–biotin–peroxidase complex (ABC) in immunoperoxidase technique: a comparison between ABC and unlabeled antibody (PAP) procedures. *J Histochem Cytochem* **29**: 577–580

Kern SE, Kinzler KW, Bruskin A, Jarosz D, Friedman P, Prives C and Vogelstein B (1991) Identification of p53 as a sequence-specific DNA-binding protein. *Science* **252**: 1708–1711

Kuerbitz SJ, Plunkett BS, Walsh WV and Kastan MB (1992) Wild-type p53 in a cell cycle checkpoint determinant following irradiation. *Proc Natl Acad Sci USA* **89**: 7491–7495

Lee JS, Yoon A, Kalapurakal SK, Ro JR, Lee JJ, Tu N, Hittelman N and Hong WK (1995) Expression of p53 oncoprotein in non-small cell lung cancer: a favorable prognostic factor. *J Clin Oncol* **13**: 1893–1903

Loechler E, Green C and Essigmann J (1984) In vivo mutagenesis by $O^6$-methylguanine built into a unique site in a viral sequence. *Proc Natl Acad Sci USA* **81**: 6271–6275

Milner J (1995) Flexibility: the key to p53 function?. *TIBS* **20**: 49–51

Mitsudomi T, Oyama T, Kusano T, Osaki T, Nakanishi R and Shiravakusa T (1993) Mutations of the *p53* gene as a predictor of poor prognosis in patients with non-small cell lung cancer. *J Natl Cancer Inst* **85**: 2018–2023

Momand J, Zambetti GP, Olson DC, George DL and Levine AJ (1992) The *mdm-2* oncogene product forms a complex with the p53 protein and inhibits p53-mediated transactivation. *Cell* **69**: 1237–1245

Mountain CF (1986) A new international staging system for lung cancer. *Chest* **89** (Suppl.): 225–233

Orita M, Suzuki Y, Sekiya T and Hayashi K (1989) Rapid and sensitive detection of point mutations and DNA polymorphisms using the polymerase chain reaction. *Genomics* **5**: 874–879

Passlick B, Izbicki JR, Riethmüller G and Pantel K (1994) p53 in non-small cell lung cancer. *J Natl Cancer Inst* **86**: 801–802

Passlick B, Izbicki JR, Häussinger K, Thetter O and Pantel K (1995) Immunohistochemical detection of p53 protein is not associated with a poor prognosis in non-small cell lung cancer. *J Thorac Cardiovasc Surg* **109**: 1205–1211

Quinlan DC, Davidson AG, Summers CL, Warden HE and Doshi HM (1992) Accumulation of p53 protein correlates with a poor prognosis in human lung cancer. *Cancer Res* **52**: 4828–4831

Ryberg D, Kure E, Lystad S, Skaug V, Stangeland L, Mercy I, Borrensen AL and Haugen A (1994) *p53* mutations in lung tumors: relationship to putative susceptibility markers for cancer. *Cancer Res* **54**: 1551–1555

Sanger F, Nicklen S and Coulson AR (1977) DNA sequencing with chain terminating inhibitors. *Proc Natl Acad Sci USA* **74**: 5463–5467

Scheffner M, Bruce AW, Huibregtse JM, Levine AJ and Howley PM (1990) The E6 oncoprotein encoded by human papillomavirus types 16 and 18 promotes the degradation of p53. *Cell* **63**: 1129–1136

Seshadri R, Leong AS, McCaul K, Firgaira FA, Setlur V, Horsfall DJ (1996) Relationship between *p53* gene abnormalities and other tumour characteristics in breast cancer prognosis. *In J Cancer* **69**: 135–141

Shipman R, Schraml P, Colombi M, Raefle G, Dalquen P and Ludwig C (1996) Frequent *TP53* gene alterations (mutation, allelic loss, nuclear accumulation) in primary non-small cell lung cancer. *Eur J Cancer* **32A**: 335–341

Smith ML, Chen IT, Zhan Q, Bae I, Chen CY, Gilmer TM, Kastan MB, O'Connor PM and Fornace AJ (1995) Involvement of the p53 tumor suppressor in repair of U.V.-type DNA damage. *Oncogene* **10**: 1053–1059

Sobin LH (1982) The World Health Organization's histological classification of lung tumors: a comparison of the first and second editions. *Cancer Detect Prev* **5**: 391–406

Top B, Mooi WJ, Klauer SG, Boerrigter L, Wisman P, Elbers M, Visser S and Rodenhuis S (1995) Comparative analysis of *p53* gene mutations and protein accumulation in human non-small cell lung cancer. *Int J Cancer* **64**: 83–91

Travis WD, Travis LB and Devesa SS (1995) Lung cancer. *Cancer* **75**: 191–202

Ullrich SJ, Anderson CW, Mercer WE and Appella E (1992) The p53 tumor suppressor protein, a modulator of cell proliferation. *J Biol Chem* **267**: 15259–15262

© Cancer Research Campaign 1997